# Predictive circulating biomarkers of the response to anti‐PD‐1 immunotherapy in advanced HER2 negative breast cancer

**DOI:** 10.1002/ctm2.70255

**Published:** 2025-02-25

**Authors:** Yuhan Wei, Hewei Ge, Yalong Qi, Cheng Zeng, Xiaoying Sun, Hongnan Mo, Fei Ma

**Affiliations:** ^1^ Department of Medical Oncology, National Cancer Center/National Clinical Research Center for Cancer/Cancer Hospital Chinese Academy of Medical Sciences and Peking Union Medical College Beijing China; ^2^ Department of Medical Oncology Cancer Hospital of HuanXing ChaoYang District Beijing China

**Keywords:** breast cancer, CyTOF, immunotherapy, PD‐1, peripheral blood mononuclear cell, predictive biomarkers, systemic immunity

## Abstract

**Background:**

Immunotherapy shows promise for treating advanced breast cancer, but only a few patients could respond. Predictive biomarkers from peripheral blood are urgently needed.

**Methods:**

We designed a comprehensive 42‐marker mass cytometry panel to profile the peripheral blood samples from 57 patients diagnosed with advanced HER2‐negative breast cancer receiving anti‐PD‐1 combination therapy. Patients were categorized as responders and non‐responders according to 6‐month progression‐free survival (PFS), followed by phenotypic and functional comparations to identify candidate predictive biomarkers. Longitudinal analysis of paired samples further revealed dynamic changes in these specific subpopulations.

**Results:**

Non‐responders exhibited significantly higher frequencies of CD39+ Tregs (adjusted *p* = .031) in the T‐cell milieu at baseline, which exhibited a positive correlation with PD‐1+ T cells in the NR group. Longitudinal assessment indicated a significant decrease of PD‐1+ T cells and an increase of CD39+ Tregs following anti‐PD‐1 treatment, suggesting their potential role in immunotherapy resistance. In the myeloid compartment, responders showed significantly higher CCR2+ monocyte‐derived dendritic cell frequencies than non‐responders (adjusted *p* = .037). These cells were positively correlated with other dendritic cells in responders but negatively with naïve T cells in non‐responders. Based on these two efficacy‐related biomarkers, we developed an immunotherapy prognostic prediction model and confirmed its superiority in distinguishing patient PFS (*p* < .001).

**Conclusion:**

Peripheral CD39+ Tregs and monocyte‐derived dendritic cells are correlated with immunotherapy response, serving as potential biomarkers to guide therapeutic choices in immunotherapy.

**Key points:**

- CD39+ Tregs in peripheral blood are associated with poor response to anti‐PD‐1 immunotherapy in advanced breast cancer.
- Higher frequencies of CCR2+ monocyte‐derived dendritic cells correlate with better immunotherapy outcomes.
- A predictive model based on CD39+ Tregs and monocyte‐derived dendritic cells effectively distinguishes patient progression‐free survival.
- Peripheral blood biomarkers offer a non‐invasive approach to guide immunotherapy choices.

## Graphical abstract

Our study identified that the proportions of CD39+ Tregs and moDCs in the peripheral blood were correlated with the response to immunotherapy in patients with advanced breast cancer. These two subpopulations are potential predictive biomarkers for determining patient prognosis, offering valuable guidance for treatment strategies.

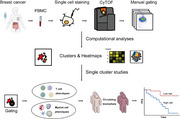

## 1 INTRODUCTION

Inhibitors targeting programmed death‐1 (PD‐1)/programmed death ligand‐1 (PD‐L1) have transformed the treatment landscape for various malignancies, including advanced human epidermal growth factor receptor 2 (HER2) negative breast cancer, demonstrating significant clinical efficacy and long‐term survival benefits.^1^, ^2^, ^3^, ^4^ However, considerable variability in patient responses remains a major challenge, as not all patients benefit from these therapies.^5^ Therefore, identifying reliable predictive biomarkers that can differentiate responders from non‐responders prior to treatment initiation is crucial for optimizing therapeutic outcomes.^6^, ^7^

The role of systemic immunity in cancer therapy has garnered significant attention, with the regulation of systemic immunity shown to be indispensable for achieving durable responses to immunotherapy.^8^, ^9^, ^10^ Peripheral blood, as a reservoir and significant conduit of systemic immunity, has emerged as a promising approach for developing predictive biomarkers, given its accessibility and potential to reflect systemic immune dynamics.^9^ Multiple studies have explored the predictive role of immune biomarkers from peripheral blood on the efficacy of immunotherapy, including protein expressions in the plasma or serum circulation,^11^, ^12^ peripheral cellular biomarkers,^13^, ^14^ and circulating tumour DNA.^15^, ^16^ However, no systemic immune biomarker has been adequately validated to facilitate decision‐making yet, particularly for cold tumours like advanced breast cancer.

In this investigation, we analyzed peripheral blood mononuclear cells (PBMCs) before and after treatment in individuals with advanced HER2‐negative breast cancer undergoing anti‐PD‐1 therapy. Utilizing cytometry by time‐of‐flight (CyTOF), we extensively characterized various cell subsets, activation markers, cytokines, and cytotoxicity markers to investigate potential correlations and dynamic changes between patients’ baseline immune profiles and the efficacy of immunotherapy, providing important insights for guiding clinical decisions in immunotherapy.

## 2 METHODS

### 2.1 Study design and prospective sample collection

Blood samples were obtained from participants engaged in a prospective randomized adaptive clinical trial (NCT04389073). Eligible subjects possessed histologically verified HER2‐negative metastatic breast carcinoma and had previously undergone no more than a single regimen of standard chemotherapy. Participants were randomly assigned via Bayesian adaptive randomization to receive toripalimab (PD‐1 inhibitor) combined with the following regimens: (1) bevacizumab + vinorelbine (NVB) (*n* = 15); (2) conventional cisplatin + NVB (*n* = 17); (3) metronomic cyclophosphamide + capecitabine + NVB (VEX cohort; *n* = 25). Specific drug administration strategies and the results of the clinical trial have been previously reported.^17^ The study received endorsement from the Ethical Committee of the National Cancer Center/Cancer Hospital, Chinese Academy of Medical Sciences, and adhered to all pertinent ethical guidelines. All patients finished written consent prior to participation.

Peripheral blood (4 mL per person) was collected from each subject in EDTA‐coated vacutainer tubes at three distinct intervals: prior to intervention, following two cycles of treatment, and upon disease progression.

### 2.2 Cell suspension preparations

Blood specimens were promptly conveyed to the laboratory for analysis. PBMCs were isolated with Ficoll utilizing density gradient centrifugation. The cell pellet was reconstituted in 5 mL of pre‐chilled phosphate‐buffered saline (PBS) and subsequently centrifuged at 400×*g* at 4°C for 5 min. The supernatant was eliminated, cells were counted, and single‐cell suspensions were cryogenically preserved within a cell‐freezing medium containing dimethyl sulfoxide in liquid nitrogen for large‐scale cell staining.

### 2.3 Antibody staining and data acquisition for CyTOF

Purified antibodies were labelled with designated metal tags utilizing the MaxPAR antibody labelling kit (Fluidigm), and calibrated to appropriate concentrations prior to application. Comprehensive details regarding each antibody are delineated in Table S1.

Single‐cell suspensions were thawed and the cells were counted. Samples designated for analysis adhered to the following quality parameters: cell count of no less than 3 × 10^6^ and viability greater than 85%.

Cells were subjected to a washing procedure utilizing 1× PBS and subsequently stained with 100 µL of 250 nM cisplatin (Fluidigm) for 5 min to eliminate non‐viable cells. Subsequently, they were subjected to an Fc receptor blockade solution and a cocktail of surface antibodies on ice for 30 min. The cells underwent two washing cycles with FACS buffer (1 × PBS + 0.5% BSA) and were subsequently fixed overnight in 200 µL of intercalation solution (Maxpar Perm and Fix Buffer infused with 250 nM 191/193Ir, Fluidigm). Post‐fixation, cells underwent a single wash with FACS buffer, followed by a wash with perm buffer (eBioscience). Subsequently, they were subjected to staining with a cocktail of intracellular antibodies for approximately 30 min. Following this, the cells were rinsed and resuspended in a deionized aqueous solution, to which 20% EQ beads (Fluidigm) were added, and data were procured utilizing a mass cytometer (Helios, Fluidigm).

### 2.4 Clustering and dimension reduction

Data from each specimen was debarcoded from raw data employing a doublet‐filtering protocol with distinct mass‐tagged barcodes. Each.fcs file produced from various batches underwent normalization via the bead normalization technique. Manual gating was performed utilizing FlowJo software to eliminate debris, non‐viable cells, and doublets, thereby isolating viable, single cells (Figure S1A).

Further manual gating was used to delineate cell subpopulations of interest, including immune cells, T lymphocytes, and myeloid cells, followed by the X‐shift clustering algorithm to classify cells into different phenotypes on the basis of the expression levels of specific markers. Dimensionality reduction using t‐SNE was employed to represent high‐dimensional data in a two‐dimensional format, illustrating the distribution of each cluster and marker expression, as well as variations between groups. The cell type of each cluster was annotated on heatmaps in accordance with its marker expression profile.

### 2.5 CyTOF statistical analysis

Independent *t*‐test or Wilcoxon test was employed, contingent upon the normality of data distribution assessed by the Shapiro–Wilk test, to evaluate the differences in both cell cluster frequencies and protein expression intensities between the R and NR groups. Dynamic changes before and after immunotherapy were explored utilizing paired *t*‐tests or paired Wilcoxon test, based on the normality of the data. *p*‐values were modified for multiple comparisons among different clusters via the Benjamini‐Hochberg (BH) methodology to regulate the false discovery rate. For patients possessing paired samples at disease progression, we conducted Friedman tests, subsequently post hoc pairwise comparisons via the Dunn‐Bonferroni method to explore dynamic changes in cell subsets and molecules of interest across three time points. Additionally, Spearman tests were used to measure the correlation between cluster abundances in paired data.

### 2.6 Construction of the prognostic model

Significant differential markers predictive of treatment efficacy were incorporated into a multivariate Cox regression model to test their prognostic value for progression‐free survival (PFS). The model's predictive performance was evaluated by the area under the receiver operating characteristic curve (AUC). After that, the optimal cut‐off value was identified by maximizing the statistical significance of the log‐rank test, which classified patients into high‐risk and low‐risk cohorts and subjected them to Kaplan–Meier survival analysis.

### 2.7 External validation

On the basis of the coefficients derived from the model, we tested it using a fully independent single‐cell dataset (GSE189125) from the Gene Expression Omnibus (GEO) database. Dimension reduction and unsupervised clustering were executed in accordance with the established protocol within the Seurat framework.^18^ After two rounds of clustering, we examined the gene expression corresponding to our cyTOF markers within each cluster to identify the cell subsets of interest for model validation, using the AUC of response as the evaluation criterion. A more comprehensive methodological section on validation set training is shown in the Supporting Information Methods.

### 2.8 Statistical analysis

Quantitative variables were expressed and presented as median values, while categorical variables were delineated as proportions. Fisher's exact test was employed for comparisons between baseline clinicopathological parameters and the two patient groups. Survival outcomes were assessed utilizing the Kaplan–Meier method, with inter‐group differences scrutinized through the log‐rank test. All statistical evaluations and visualizations were performed utilizing R (version 4.4.0). A bidirectional *p*‐value of less than.05 was regarded as statistically significant.

## 3 RESULTS

### 3.1 Identification of immune cells associated with the effectiveness of immunotherapy in HER2‐negative breast cancer patients

**FIGURE 1:**
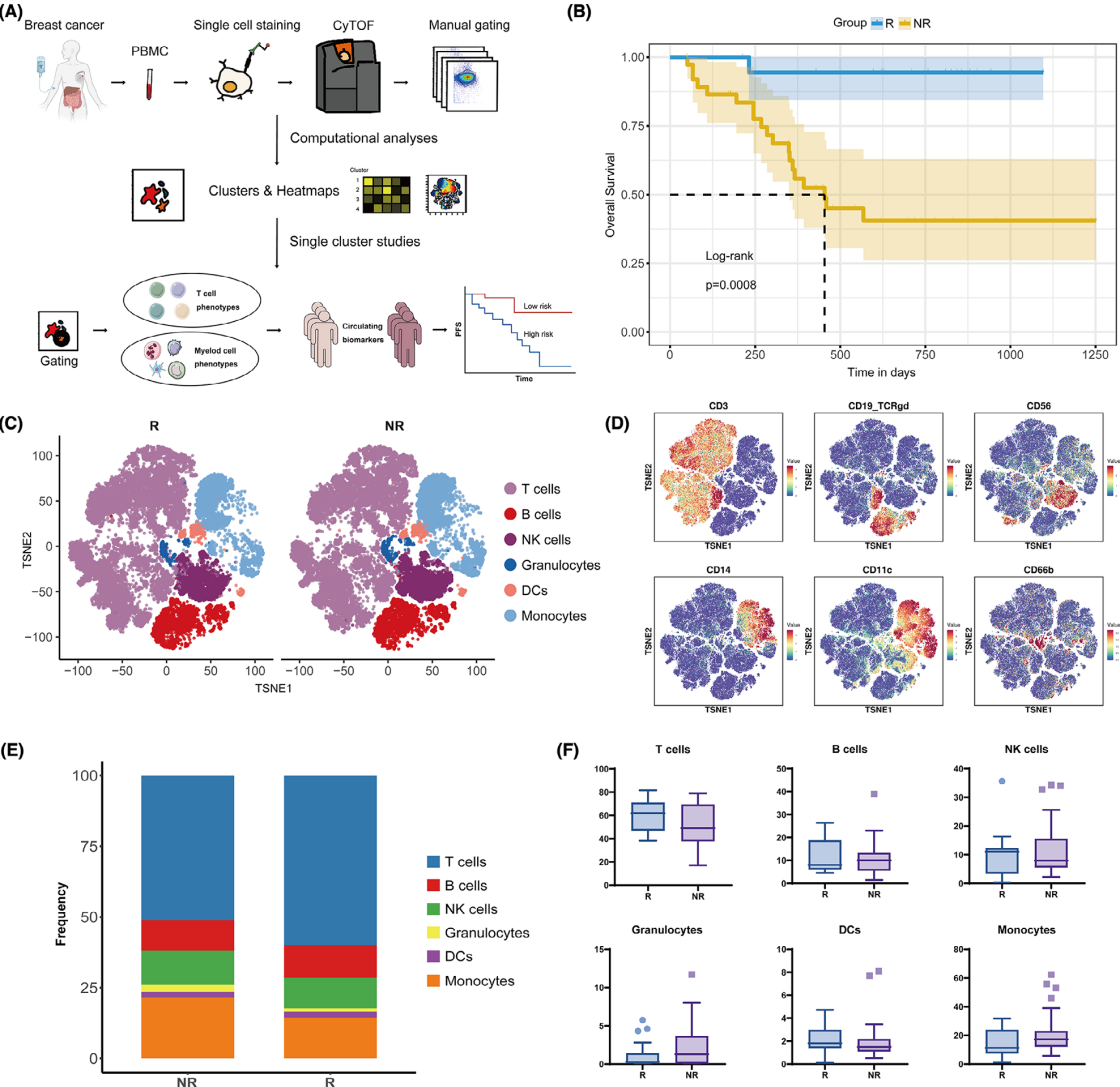
Mass cytometry analysis reveals the immune cell atlas in patients with advanced breast cancer. (A) Schematic diagram of the study. (B) Kaplan–Meier survival analysis of overall survival in the responder (R) and non‐responder (NR) groups. (C) tSNE maps displaying the distribution of main immune cells between the two groups. (D) The expression of the lineage markers for the identification of the main types of immune cells. (E, F) Bar and box plots showing the differences in the proportions of major immune cell subsets between the two groups.

A total of 57 individuals diagnosed with advanced HER2‐negative breast cancer and underwent treatment with PD‐1 inhibitor combined with chemotherapy participated in this study (NCT04389073).^17^ Patients were categorized into two groups based on their 6‐month PFS: responders (R) exhibiting a PFS of 6 months or greater, and non‐responders (NR) with a PFS of less than 6 months. A comprehensive examination of immune cell subpopulations was subsequently conducted between the two cohorts (Figure 1A). The initial appraisal of the baseline clinicopathological revealed no significant correlation between the response groups and these characteristics (Table 1). Patients in the R group had significantly better PFS and overall survival (Figure 1B; Figure S1B).

**TABLE 1:** Baseline clinical characteristics of patients with metastatic breast cancer.

Characteristics	R (PFS≥6 m) *N* = 19	NR (PFS < 6 m) *N* = 38	*p‐*value
Age			.247
Medium	51 (30–76)	50 (28–73)	
<40	1 (5.3)	8 (21.1)	
>40	18 (94.7)	30 (78.9)	
ECOG			.379
0	11 (57.9)	27 (71.1)	
1	8 (42.1)	11 (28.9)	
Subtype			1.000
HR+HER2‐	9 (47.4)	19 (50)	
TNBC	10 (52.6)	19 (50)	
HER2			.244
Low	11 (57.9)	28 (73.7)	
Zero	8 (42.1)	10 (26.3)	
PD‐L1 expression			.592
Negative	2 (40)	5 (62.5)	
Positive	3 (60)	3 (37.5)	
Unclear	14	30	
Line			1.000
1	10 (52.6)	19 (50)	
2	9 (47.4)	19 (50)	
Regimens			.286
Toripalimab+NVB+BEV	3 (15.8)	12 (31.6)	
Toripalimab+NVB+DDP	5 (26.3)	12 (31.6)	
Toripalimab+VEX	11 (57.9)	14 (36.8)	
Efficacy			<.001
PR	8 (42.1)	6 (15.8)	
SD	11 (57.9)	14 (36.8)	
PD	0 (0)	18 (47.4)	
Liver_metastases			.236
No	15 (78.9)	23 (60.5)	
Yes	4 (21.1)	15 (39.5)	
Lung_metastases			.576
No	11 (57.9)	18 (47.4)	
Yes	8 (42.1)	20 (52.6)	
Lymph_node_metastases			.576
No	11 (57.9)	18 (47.4)	
Yes	8 (42.1)	20 (52.6)	
Bone_metastases			.574
No	12 (63.2)	20 (52.6)	
Yes	7 (36.8)	18 (47.4)	
Brain_metastases			1.000
No	17 (89.5)	35 (92.1)	
Yes	2 (10.5)	3 (7.9)	
Chest_wall_metastases			.343
No	16 (84.2)	27 (71.1)	
Yes	3 (15.8)	11 (28.9)	
Pleura_metastases			.652
No	18 (94.7)	33 (86.8)	
Yes	1 (5.3)	5 (13.2)	

Abbreviations: R, responders; NR, nonresponders; HER2, human epidermal growth factor receptor 2; PD‐L1, programmed cell death ligand‐1.

PBMCs were collected from the patients and analyzed via CyTOF to detect various immune cell subpopulations. Following quality filtering and marker identification, we identified multiple immune cell subsets, encompassing T lymphocytes, B lymphocytes, natural killer (NK) lymphocytes, granulocytes, dendritic cells (DCs), and monocytes (Figure 1C,D; Figure S1C). Consistent with previous reports,^17^ responders exhibited a tendency for a higher proportion of T cells (*p* = .082; adjusted *p* = .257) and a diminished prevalence of monocytes (*p* = .086; adjusted *p* = .257) in the peripheral blood, but these disparities did not attain statistical significance (Figure 1E,F; Table S2).

To elucidate the specific immune cell subsets that were predictive of therapeutic efficacy, we manually gated the two primary immune cell subpopulations in the peripheral circulation: T lymphocytes and myeloid cells, followed by clustering and dimensionality reduction for further in‐depth analysis.

### 3.2 Activated Tregs are robust predictors of poor clinical outcomes in immunotherapy

**FIGURE 2:**
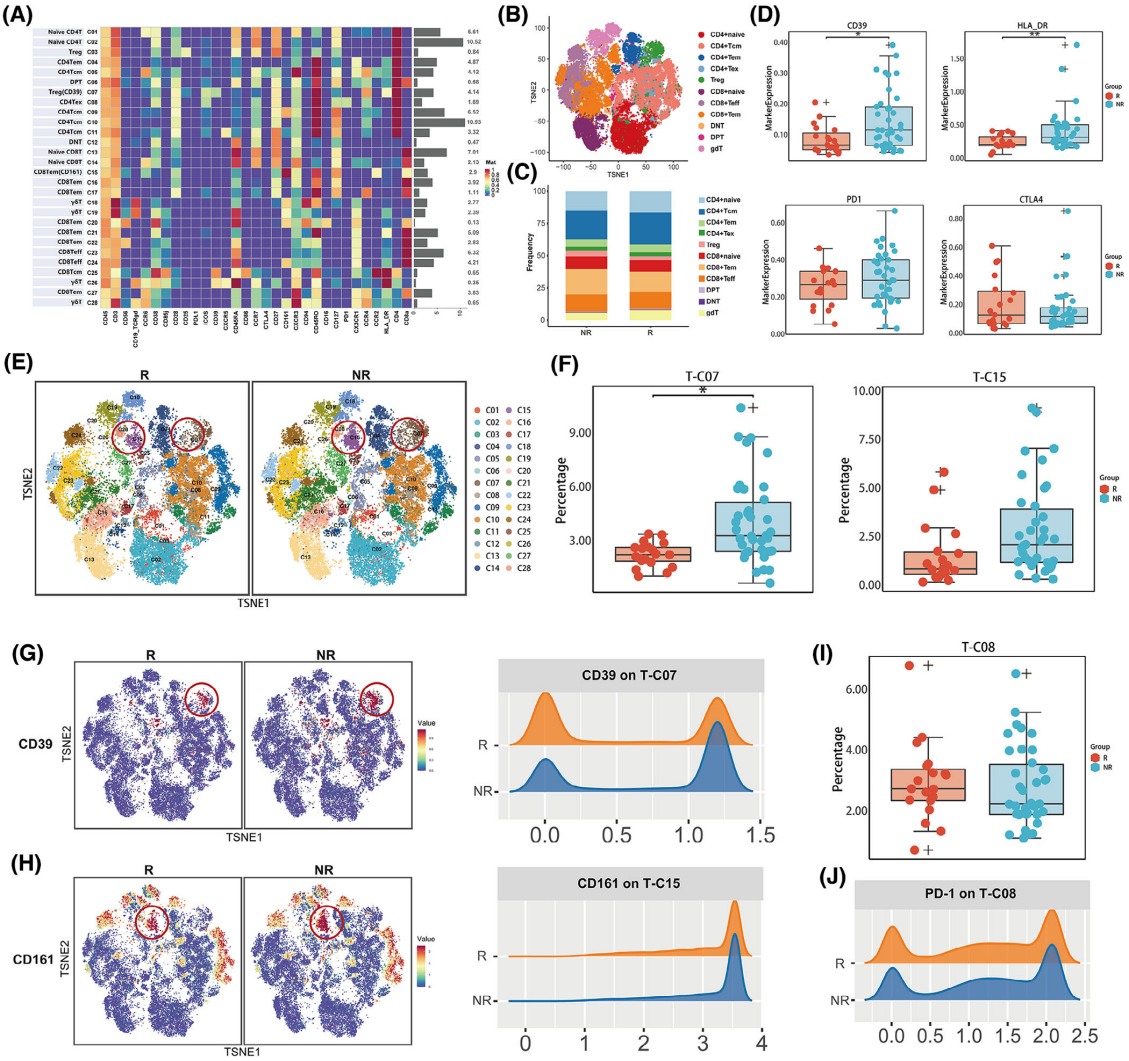
Mass cytometry and marker expression analysis of the T‐cell compartment. (A) Heatmap displaying a normalized expression of the 42 pre‐designed markers for the 28 clusters. (B) tSNE maps displaying the distributions of main T‐cell subsets. (C) Proportions of the main T cells in patients in different groups. (D) Differential marker expression on T cells in responders (R) and non‐responders (NR). (E, F) Proportions of T‐cell subsets in clusters 7 (CD39+ Treg) and 15 (CD161+CD8+Tem) differed between the two groups. (G, H) Comparison of the expression of CD39 on T‐C07 (top) and CD161 on T‐C15 (bottom) between the two groups. (I, J) Proportions of T‐C08 (PD‐1+ CD4+ Tex) and PD‐1 expression on T‐C08 in the two groups.

Given the pivotal function of T lymphocytes in immunotherapy, we first manually gated the T‐cell compartment based on CD3 expression. Using unsupervised clustering and manual annotation, we identified 28 T‐cell subpopulations, which included CD4+ T cells and CD8+ T cells of various differentiation stages, such as naïve T (Tn), central memory T (Tcm), effector memory T (Tem), effector T (Teff), exhausted T (Tex) and regulatory T (Treg) cells, as well as double‐positive T (DPT), double‐negative T (DNT), and gdT cells (Figure 2A,B; Figure S2A). Overall, responders tended to have more CD4+ Tcm cells, concomitantly presenting with fewer Tregs and CD8+ Tem cells (Figure 2C). In terms of marker expression across all T cells, non‐responders exhibited significantly increased levels of CD39 and HLA‐DR, whereas the expression levels of PD‐1 and cytotoxic T lymphocyte antigen‐4 (CTLA‐4) were not notably different (Figure 2D; Table S3).

Further analysis of specific cell subpopulations revealed that, compared with responders, non‐responders had higher baseline levels of T‐C07 (CD39+ Treg) (*p* < .001; adjusted *p* = .031) and T‐C15 (CD161+ CD8+ Tem) (*p* = .006; adjusted *p* = .097) (Figure 2E,F; Table S4). These two subpopulations demonstrated similar trends across all three treatment cohorts (Figure S2B). In addition, these patients also presented increased expression of CD39 on CD39+ Treg cells and CD161 on CD161+ CD8+ Tem cells (Figure 2G,H).

In particular, we examined the correlation between PD‐1 expression and treatment efficacy. A subset of PD‐1+ CD4+ T cells (T‐C08), termed CD4+ Tex, was identified. The baseline frequency of this population tended to be higher in responders, but without statistical significance (*p* = .377; adjusted *p* = 1) (Figure 2I). Additionally, the intensity of PD‐1 expression in these cells was also comparable between the two cohorts (Figure 2J). Other T‐cell subpopulations and surface marker expressions were not significantly associated with efficacy (Figure S2C,D; Table S4).

Our data suggest a potential association between elevated levels of CD39+ Tregs within the T‐cell milieu and inferior outcomes in patients undergoing anti‐PD‐1 therapy, indicating promising predictive potential for treatment efficacy.

### 3.3 The immunosuppressive T cells cannot be blocked by PD‐1 inhibitors, leading to primary resistance

**FIGURE 3:**
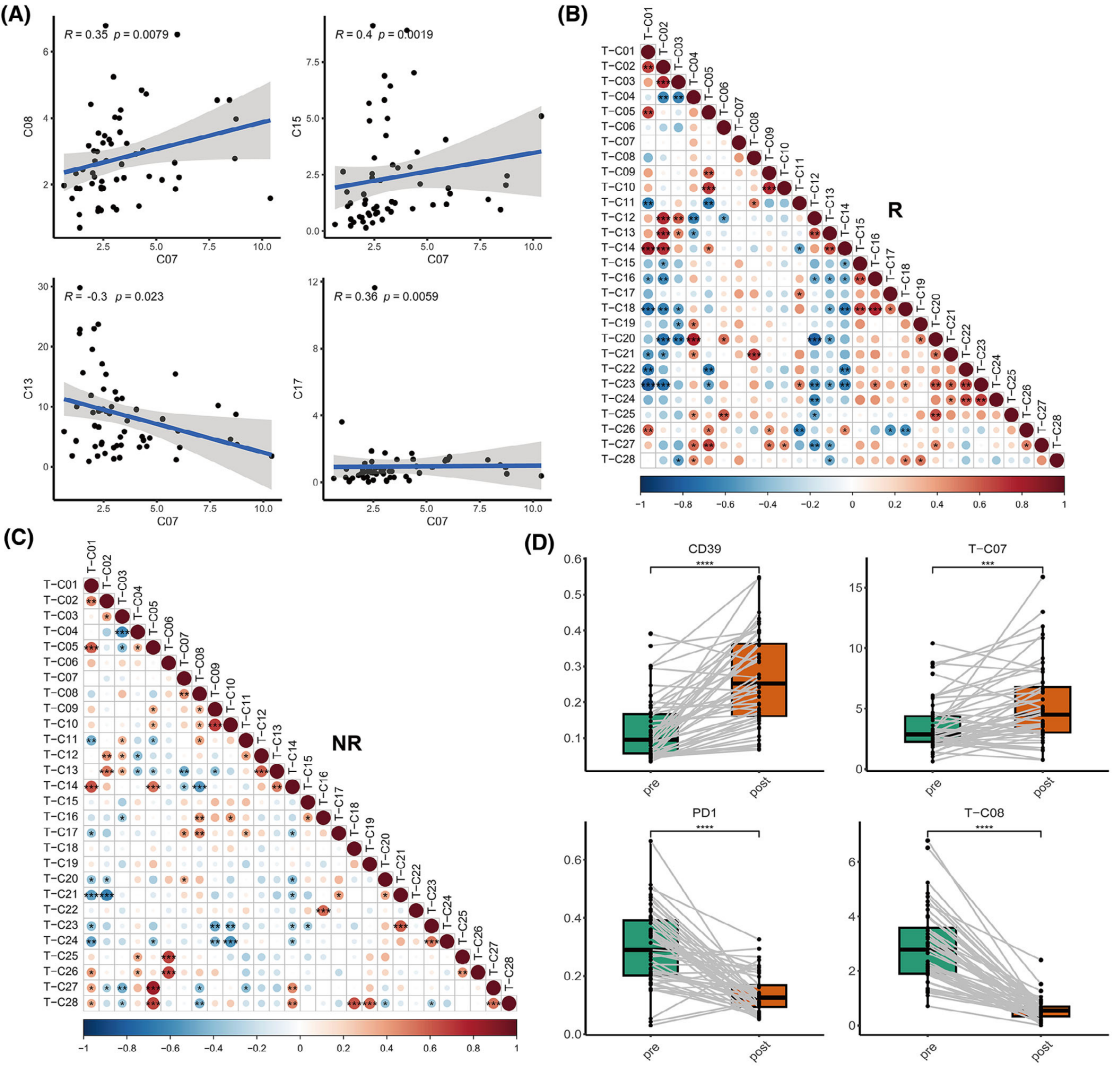
Correlation and longitudinal analysis of the specific immunosuppressive T‐cell subsets. (A) Correlations between the two specific T‐cells and other T‐cell subsets with significance for all patients. (B, C) Correlations among different T‐cell clusters in responders (R) and non‐responders (NR), respectively. (D) Longitudinal analysis of the expression of CD39 and PD‐1 as well as the proportions of T‐C07 and T‐C08.

Correlation analyses among different T‐cell subpopulations were conducted to obtain a more profound understanding of the possible immunosuppressive mechanisms exhibited by these cells (Figure S3A). It was revealed that the prevalence of T‐C07 (CD39+ Treg) exhibited a significant positive correlation with that of T‐C15 (CD161+ CD8+ Tem), and negatively correlated with that of C13 (naïve CD8+ T cells) (Figure 3A; Figure S3A). Notably, these negative relationships were primarily observed in the NR group (Figure 3B,C). Moreover, a significant negative correlation was detected between T‐C15 (CD161+ CD8+ Tem) and T‐C23 (CD8+ Teff) within the NR group, whereas such a relationship was absent in the R group (Figure 3B,C). These findings confirm that the activated immunosuppressive T‐cell subpopulations may influence both naïve CD8+ T cells and CD8+ Teff in systemic immunity.

Interestingly, we found that in the NR group, T‐C07 (CD39+ Tregs) exhibited a markedly positive correlation with T‐C08 (PD‐1+ CD4+ T cells), and T‐C15 also showed a positive correlation trend with T‐C08 (Figure 3C). These findings indicate that these cells may collaboratively exert immunosuppressive effects along with PD‐1+ T cells.

To elucidate the causes of resistance to immunotherapy, we further analyzed the dynamic changes (baseline, after two cycles of treatment) of the immune landscape in the peripheral blood of these patients in these immune cell subpopulations in peripheral blood. Among them, paired blood samples at the progression stage were also procured from nine patients to further explore their potential roles in acquired resistance. We found that the overall expression of PD‐1 on T cells and the frequency of PD‐1+ CD4+ Tex (T‐C08) significantly decreased after treatment and at progression compared with baseline, indicating the effect of PD‐1 inhibitors (Figure 3D; Figure S3B; Tables S5 and S6). However, the expression of CD39 and the frequency of T‐C07 (CD39+ Tregs) significantly increased after two cycles of treatment (Figure 3D; Figure S3C; Tables S5 and S6). CD161 expression and T‐C15 did not notably change after treatment (Figure S3C; Tables S5 and S6). These findings suggest that these patients might not benefit from inhibiting the PD‐1/PD‐L1 pathway alone and may require additional targeted therapies, such as antagonists of CD39 and CD161, to achieve better outcomes.

### 3.4 CCR2+ monocyte‐derived dendritic cells (moDCs) in the myeloid compartment are associated with immunotherapy responses

**FIGURE 4:**
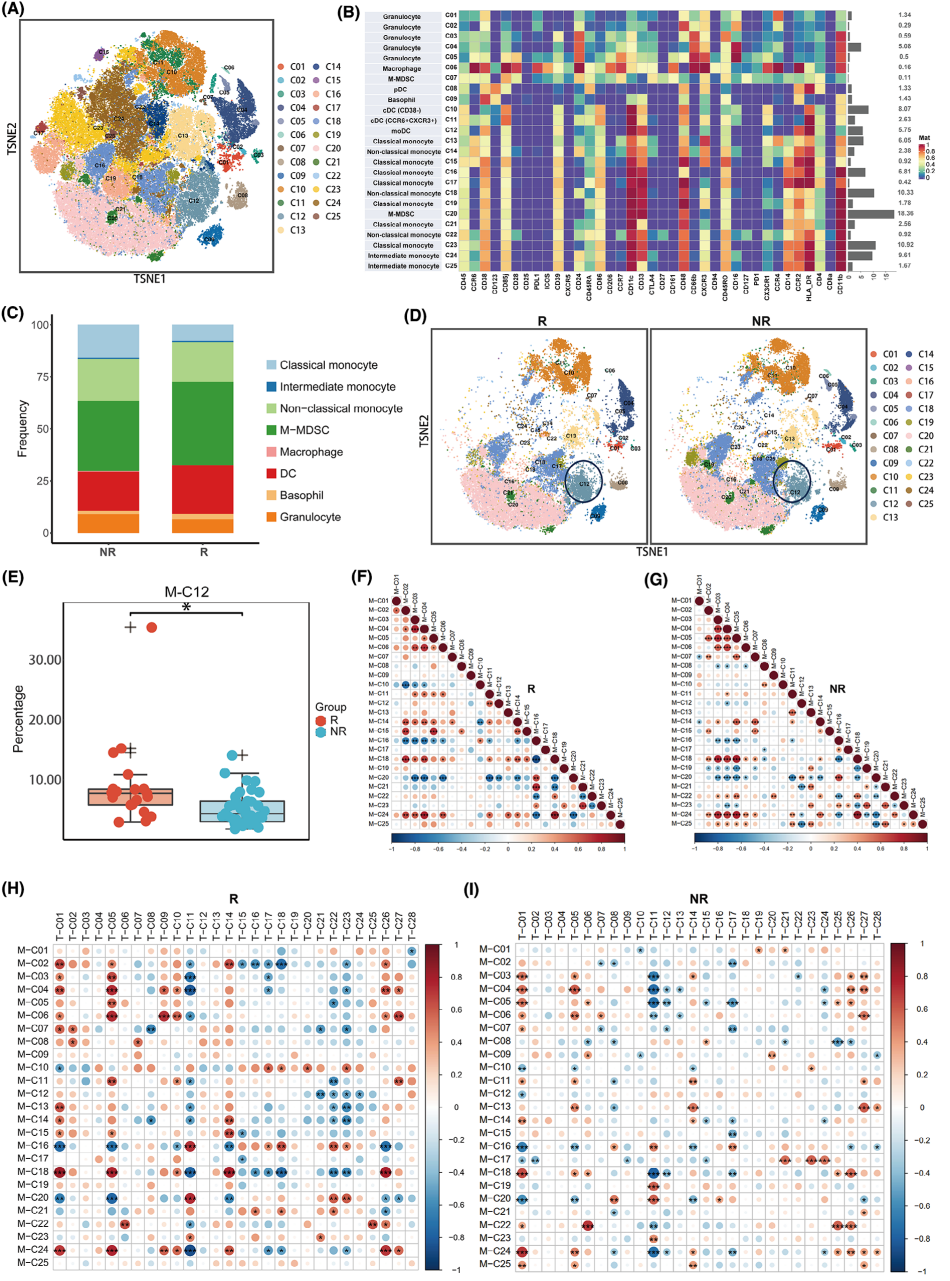
Mass cytometry of the myeloid cell compartment. (A, B) tSNE map and heatmap displaying the distribution and marker expression of the 25 myeloid cell clusters. (C) Bar plots showing the differences in the proportions of major myeloid cell subsets between responders (R) and non‐responders (NR). (D, E) Proportions of M‐C12 (CCR2+ moDC) in myeloid cell subsets showed differences between the two groups. (F, G) Correlations among different myeloid cell clusters in patients with the two groups, respectively. (H, I) Correlations among different T cell clusters and myeloid cell clusters in patients with the two groups, respectively.

Next, we focused on the ability of the myeloid compartment to forecast the efficacy of patients undergoing immunotherapy for advanced breast cancer. By clustering cells gated on CD11b, we identified 25 subpopulations, including DCs, macrophages, monocytes, neutrophils, and eosinophils (Figure 4A,B). Overall, responders tended to have more DCs and monocytic myeloid‑derived suppressor cells (M‐MDSCs) along with fewer classical monocytes (Figure 4C). The expression analysis in myeloid cells did not reveal any molecules with statistical significance that could predict PFS (Figure S4A; Table S7). We paid particular attention to the expression of PD‐L1, which tended to be higher in non‐responders, although this discrepancy did not reach statistical significance (*p* = .135; Figure S4B).

Within these subpopulations, the baseline levels of M‐C12 (activated CCR2+ moDCs) were found to be more pronounced in the R group compared with the NR group (*p* = .001; adjusted *p* = .037) (Figure 4D,E; Table S8). This trend was consistently observed across all three treatment cohorts (Figure S4C). Correlation analysis revealed a noteworthy positive association between this subpopulation and M‐C08 (pDCs), alongside a significant negative association with monocytes (Figure S4D,E). Separate correlation analyses demonstrated that in the R group, M‐C12 (CCR2+ moDCs) was significantly positively correlated with M‐C11 (CCR6+ CXCR3+ cDCs) (Figure 4F), which significantly increased after two cycles of treatment and at progression compared with baseline (Figure S4F; Table S9). This finding confirms that the collaboration between the two different types of DCs in the peripheral circulation may serve as a crucial determinant in the effectiveness of immunotherapeutic approaches. In contrast, in the NR group, M‐C12 was significantly positively correlated with M‐C20 (M‐MDSCs), a population of immunosuppressive cells whose abundance significantly decreased after treatment and at progression compared with baseline (Figure 4G, Figure S4F; Table S9). These findings suggest that in the NR group, the M‐C12 population might be inhibited by MDSCs, leading to a lack of efficacy. Other myeloid cell subpopulations were not significantly related to patient efficacy (Figure S4G; Table S8).

Since myeloid cells influence anti‐tumour immunity primarily by directly or indirectly affecting T cells, we subsequently explored the relationships between myeloid cells and T‐cell subpopulations within the peripheral blood. We found that in the R group, M‐C12 (CCR2+ moDCs) was significantly negatively correlated with T‐C21 (CD8+ Tem), T‐C22 (CD8+ Tem), T‐C23 (CD8+ Teff), and T‐C24 (CD8+ Teff; Figure 4H). These four subpopulations were characterized by CD38 and CXCR3 negativity, indicating a population in a non‐chemotactic, non‐activated state inclined towards terminal differentiation. Conversely, in the NR group, the M‐C12 subpopulation was significantly negatively correlated with T‐C01 (naïve CD4+ T cells) (Figure 4I), which could be detrimental to the activation of T‐cell immunity.

### 3.5 Combined parameters in peripheral blood effectively predict PFS after immunotherapy

**FIGURE 5:**
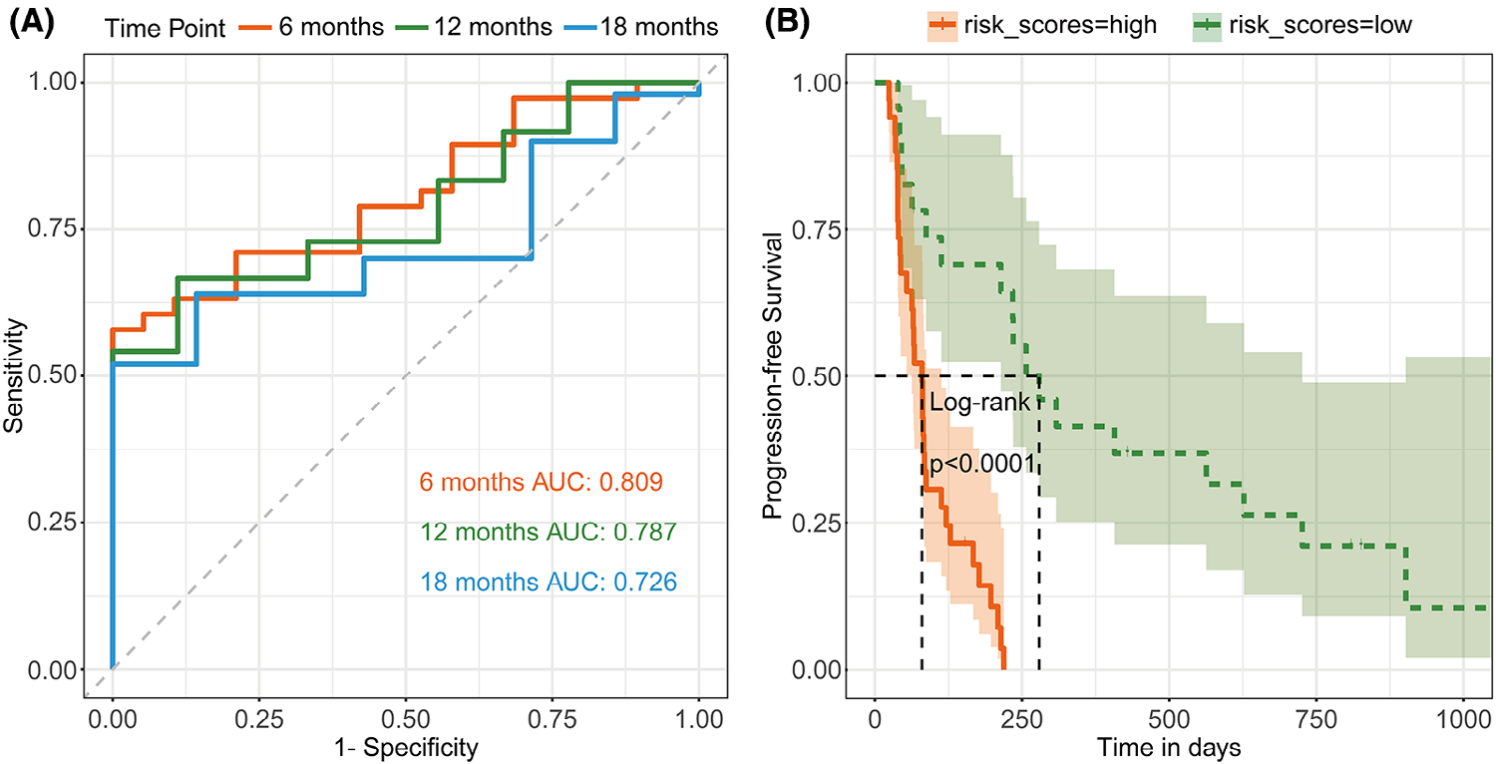
Prognostic value of “Breast Cancer Immunotherapy Predictive Score” derived from efficacy‐related biomarkers. (A) Receiver operating characteristic (ROC) curve of the model in classifying 6‐month, 12‐month, 18‐month progression‐free survival. (B) Kaplan–Meier survival analysis of progression‐free survival by the risk score groups.

We further investigated the predictive potential of these efficacy‐related immune cell subpopulations for patient PFS. By integrating the two immune cell subpopulations identified in our previous analyses (CD39+ Tregs and CCR2+ moDCs) into a predictive model named the “Breast Cancer Immunotherapy Predictive Score”, we found that this composite risk score also effectively predicted PFS, with AUC values for 6‐month, 12‐month, and 18‐month PFS of.809,.787, and.726, respectively (Figure 5A), and its predictive efficacy was independent of the treatment group (Table S10). Utilizing a risk score cut‐off of.84, we delineated PFS curves for the low‐risk and high‐risk cohorts, which distinctly differentiated the two groups regarding their PFS outcomes (log‐rank, *p* < .001; Figure 5B).

The predictive performance of the model was robustly validated across the three treatment cohorts: the cisplatin cohort (AUC values at 6, 12, and 18 months were.717,.833, and.688, respectively), the bevacizumab cohort (AUC values at 6, 12, and 18 months were.750,.808, and.808, respectively), and the VEX cohort (AUC values at 6, 12, and 18 months were.883,.720, and.619, respectively; Figure S5A).

To appraise the robustness of the model, we selected the single‐cell dataset GSE189125 for validation, which includes 14 melanoma patients administered immunotherapy.^19^ The major clusters of T and myeloid cells were first identified (Figure S5B). Subclustering and analysis of the expression signatures revealed clusters 15 and 23 in T cells correspond to T‐C07 in cyTOF analysis (Figure S5C,D), and clusters 5 and 15 in myeloid cells correspond to M‐C12 in cyTOF analysis (Figure S5E,F). Using the proportion of these subpopulations and their respective model coefficients as input, we predicted the probability of treatment response (5 non‐responders and 9 responders), which yielded an AUC of.64 within the single‐cell external test cohort (Figure S5G). These results confirm that the combined risk score is a robust prognosticator of outcomes and treatment responsiveness to immunotherapy.

Further exploratory univariate analysis revealed that five clusters (T‐C07, T‐C15, T‐C27, M‐C05, and M‐C13) could predict PFS as well, whereas common clinicopathological features failed to predict treatment efficacy (Table S11). Notably, T‐C07 emerged as an independent prognosticator of both treatment responsiveness and overall PFS, suggesting its potential role in both primary and secondary resistance to immunotherapeutic interventions.

## 4 DISCUSSION

In this study, based on a prospective clinical trial of checkpoint inhibitor therapy in advanced HER2‐negative breast cancer, we analyzed the relationship between the efficacy of immunotherapy and the composition of patient PBMCs via CyTOF analysis. According to the definition of primary resistance to immunotherapy established by the Society for Immunotherapy of Cancer (SITC) consensus,^20^, ^21^ we used 6 months to distinguish whether patients responded effectively to immunotherapy. By comparing individuals within the R and NR cohorts, we identified a subset of activated immunosuppressive T cells (CD39+ Tregs) linked to resistance against anti‐PD‐1 immunotherapy, alongside CCR2+ moDC serving as a prognostic indicator of immunotherapy benefits. The combination of these two cell subpopulations also effectively distinguished PFS in patients receiving immunotherapy, serving as a biomarker to guide immunotherapy choices for “cold tumours” like breast cancer.

Generally, Tregs are well‐known immunosuppressive T cells that implement their functions via a multitude of mechanisms. Our analysis identified two populations of Treg cells. One exhibiting an activated phenotype marked by HLA‐DR and CD39 positivity was associated with resistance to immunotherapy, whereas the other Treg population without the expression of these activation markers did not differ between the two groups. CD39 (encoded by PDCD1) exerts immunosuppressive effects on various immune cells, including Tregs, by converting ATP into the immunosuppressive ADO.^22^, ^23^, ^24^ CD39‐deficient mice exhibit impaired Treg cell suppressive characteristics both in vitro and in vivo.^25^ Sade‐Feldman noted that the presence of CD39 on tumour‐infiltrating T cells represents a dysfunctional state, which is associated with a lack of response to immune checkpoint blockade in patients with melanoma.^26^ Retseck found that diminished baseline concentrations of circulating CD39+ Tregs were markedly linked to improved relapse‐free survival after anti‐CTLA‐4 treatment in melanoma patients.^27^ Our study revealed a similar association within the peripheral circulation of patients with breast cancer receiving immunotherapy. Additionally, it was observed that these CD39+ Tregs also express CCR4. Previous studies have shown that CCR4, binding with CCL17/CCL22, can induce Treg cell enrichment within the tumour microenvironment (TME). Moreover, the application of anti‐CCR4 monoclonal antibodies significantly reduces the number of intra‐tumoral Tregs, thereby alleviating Treg‐induced tumour immunosuppression. Thus, we hypothesized that CCR4‐expressing CD39+ Tregs in peripheral blood could be chemotactically attracted to the tumour microenvironment to exert immunosuppressive effects, leading to immunotherapy resistance and a worse prognosis in our study. Moreover, we discerned a favourable association between CD39+ Tregs and PD‐1+ T cells in patients resistant to immunotherapy, suggesting their joint involvement in tumour immune evasion.

Meanwhile, we also identified another group of immunosuppressive T cells, CD161+ CD8+ Tem, as a negative prognostic factor for efficacy. However, after adjusting the *p*‐value across all T‐cell subpopulations, it lost statistical significance. CD161, encoded by the KLRB1 gene, is recognized as a significant inhibitory receptor on NK and tumour‐infiltrating T cells. It is implicated in various mechanisms such as antigen recognition, inhibition of cytotoxicity, and suppression of cytokine production within lymphocyte populations.^28^, ^29^, ^30^ Prior research has indicated that patients exhibiting elevated CD161 expression within TME have markedly reduced overall survival than those with diminished CD161 expression.^31^ Nonetheless, we also found that CD161+ CD8+ Tem positively interacts with CD39+ Treg in peripheral blood, suggesting that it may exert an immunosuppressive role in conjunction with CD39+ Treg rather than independently.

Furthermore, we found that both immunosuppressive T‐cell populations were positively associated with PD‐1+ T cells, jointly exerting immunosuppressive functions. After treatment, PD‐1+ T cells were significantly suppressed; however, these two immunosuppressive cell populations could not be effectively inhibited and even exhibited a rebound increase, leading to poor efficacy. Based on these observations, we hypothesize that the detection of these specific subpopulations in the peripheral circulation of patients may indicate the need for combined therapies targeting additional specific targets, such as CD39 or CD161 antagonists, to potentially improve treatment outcomes.

In addition to T cells, myeloid cells are another crucial subgroup influencing anti‐tumour immunity. Our study unveiled that the fraction of CCR2+ moDCs in the myeloid compartment was associated with the response to immunotherapy. DCs are the most professional antigen‐presenting cells in vivo that play a crucial role in triggering robust and durable anti‐tumour responses.^32^ In this study, we identified four distinct DC populations and found that moDCs had the strongest association with patient prognosis. Unlike other DCs, moDCs are uniquely derived from monocytes via CCR2.^33^ They usually possess high tumour antigen processing capabilities, playing a significant role in antigen processing, presentation, and cross‐presentation, but exert only moderate T cell‐stimulating abilities due to nitric oxide‐mediated immunosuppressive effects.^32^ Compared with classical DCs (cDCs), the specific role of moDCs in cancer has been less studied. Nevertheless, multiple studies across various cancers have demonstrated the significance of these in mediating anti‐tumour immune responses.^34^ Some studies also indicate that moDCs are linked to an augmented proliferation of T cells within the TME after anti‐PD‐1 therapy.^35^ In this study, we observed distinctive interactions between moDCs and other cell clusters within the R and NR cohorts. In the R group, moDCs primarily enhanced the efficacy of immunotherapy by collaborating with other DCs to activate T cells. In contrast, in the NR group, moDCs were negatively correlated with naïve T cells.

Notably, there are several constraints in this study. First, the sample size was restricted, with 57 patients included for efficacy prediction. Nevertheless, this study represents the largest attempt to forecast the effectiveness of immunotherapy based on immune cell subpopulations in peripheral blood for advanced breast cancer patients, and we also validated our main conclusions using public data as an external validation set to ensure the accuracy of the identified biomarkers. Second, since our patient samples were drawn from an adaptive randomized clinical trial that combined immunotherapy with three different chemotherapy schemes, there was some heterogeneity in the treatment cohorts. To address this, we used the 6‐month PFS as a node to minimize the short‐term effects of chemotherapy and identify patients who truly benefit from immunotherapy for durable efficacy. We also validated the performance of this approach in each treatment cohort. However, owing to the limited sample size in the treatment subgroups, the interpretation of subgroup results should be approached with caution. Additionally, although mass cytometry technology has significantly improved panel capacity compared with flow cytometry, the quantity of antibodies that can be conjugated remains limited. Consequently, our primary focus was directed towards the major subpopulations of T lymphocytes and myeloid cells within the peripheral blood, which most significantly impact immunotherapy efficacy. The predictive roles of other immune cell subpopulations, including B lymphocytes and NK cells, remain to be further explored. Lastly, since the principal objective of this study was to guide clinical choices, we focused mainly on the relationship between efficacy and the immune atlas in the peripheral blood at baseline. In fact, factors determining the overall PFS and prognosis of patients include not only primary resistance but also acquired resistance (defined as progression after 6 months),^20^, ^21^ which may involve more complex, dynamic, and secondary biological effects. Although we proposed some hypotheses through the analysis of paired samples from three longitudinal time points and referenced literature, caution is warranted in interpreting these results, given the restricted number of patients whose samples are available at all three time points, and the biological functions and specific predictive effects of these subpopulations in advanced breast cancer still require further systematic basic research and prospective validation in larger clinical trials. Based on these findings, we initiated a multicenter prospective randomized controlled trial to explore the effectiveness and safety of anti‐PD‐L1 treatment combined with chemotherapy compared with chemotherapy alone in TNBC patients (NCT06229067) and prospectively collected patient tissue and blood samples for translational research to further elucidate these biomarkers indicative of immunotherapeutic response in advanced breast cancer.

In conclusion, we demonstrated that immune monitoring of CD39+ Tregs and moDCs in the peripheral blood can predict the response to anti‐PD‐1 treatment in individuals with advanced HER2‐negative breast cancer. We developed a formula based on the proportions of these two cell subpopulations, which can function as biomarkers for forecasting therapeutic outcomes in these patients. Our findings have clinical significance as they support immunotherapy selection in clinical practice and provide a basis for new treatment strategies for patients with different systemic immune statuses.

## AUTHOR CONTRIBUTIONS

Conception and design: Fei Ma and Yuhan Wei. Acquisition of data: Fei Ma, Yuhan Wei, Yalong Qi, and Xiaoying Sun. Analysis and interpretation of data: Yuhan Wei, Hewei Ge, Hongnan Mo, and Fei Ma. Review and revision of the manuscript: Yuhan Wei, Hewei Ge, Cheng Zeng, and Hongnan Mo. Final approval of the manuscript: all authors. Fei Ma is acting as the guarantor of this work.

## CONFLICT OF INTEREST STATEMENT

The authors declare no conflict of interest.

## ETHICS STATEMENT AND CONSENT TO PARTICIPATE

The study was approved by the Ethical Committee of the National Cancer Center/Cancer Hospital, Chinese Academy of Medical Sciences (no. 19/295‐2079) and complied with all relevant ethical regulations. Participants gave informed consent to participate in the study before taking part.

## Supporting information



Supporting Information

Supporting Information

Supporting Information

## Data Availability

Data are available from the corresponding upon a reasonable request.
