# Supplementary material for: Predictive circulating biomarkers of the response to anti‐PD‐1 immunotherapy in advanced HER2 negative breast cancer
Source: Clin Transl Med. 2025 Feb 25;15(3):e70255. doi: 10.1002/ctm2.70255 (PMC11859116; doi:10.1002/ctm2.70255)
Supplement: Supplementary file 1 — Supporting Information [file CTM2-15-e70255-s003.docx]

**Predictive Circulating Biomarkers of the Response to Anti-PD-1 Immunotherapy in Advanced HER2 Negative Breast Cancer**

Yuhan Wei, Hewei Ge, Yalong Qi, Cheng Zeng, Xiaoying Sun, Hongnan Mo, Fei Ma

**Figure S1.** **Mass Cytometry Analysis Reveals the Immune Cell Atlas in advanced breast cancer Patients, related to Figure 1.**


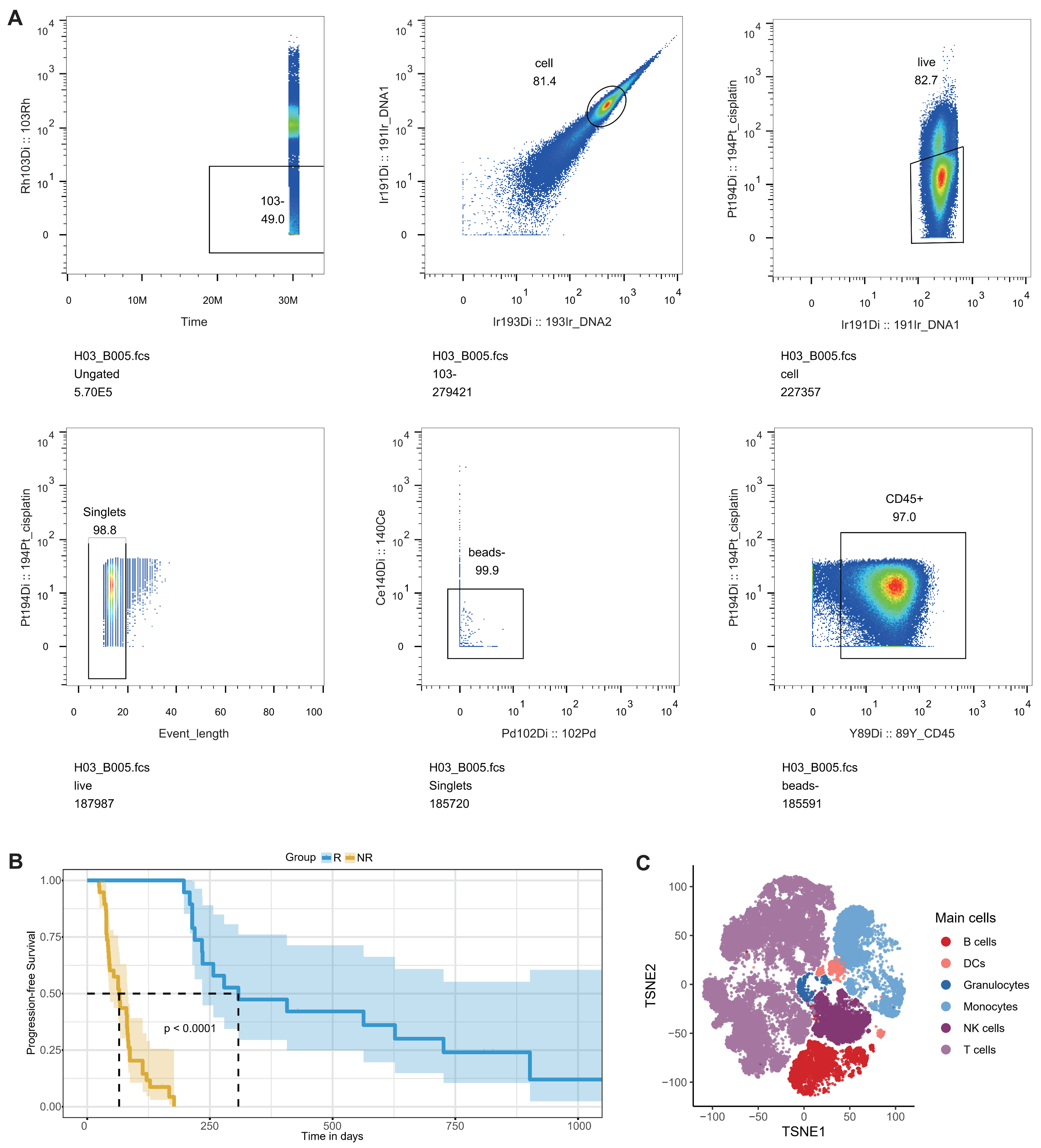


**Figure S2 In-depth analysis of T-cell compartment, related to Figure 2.**


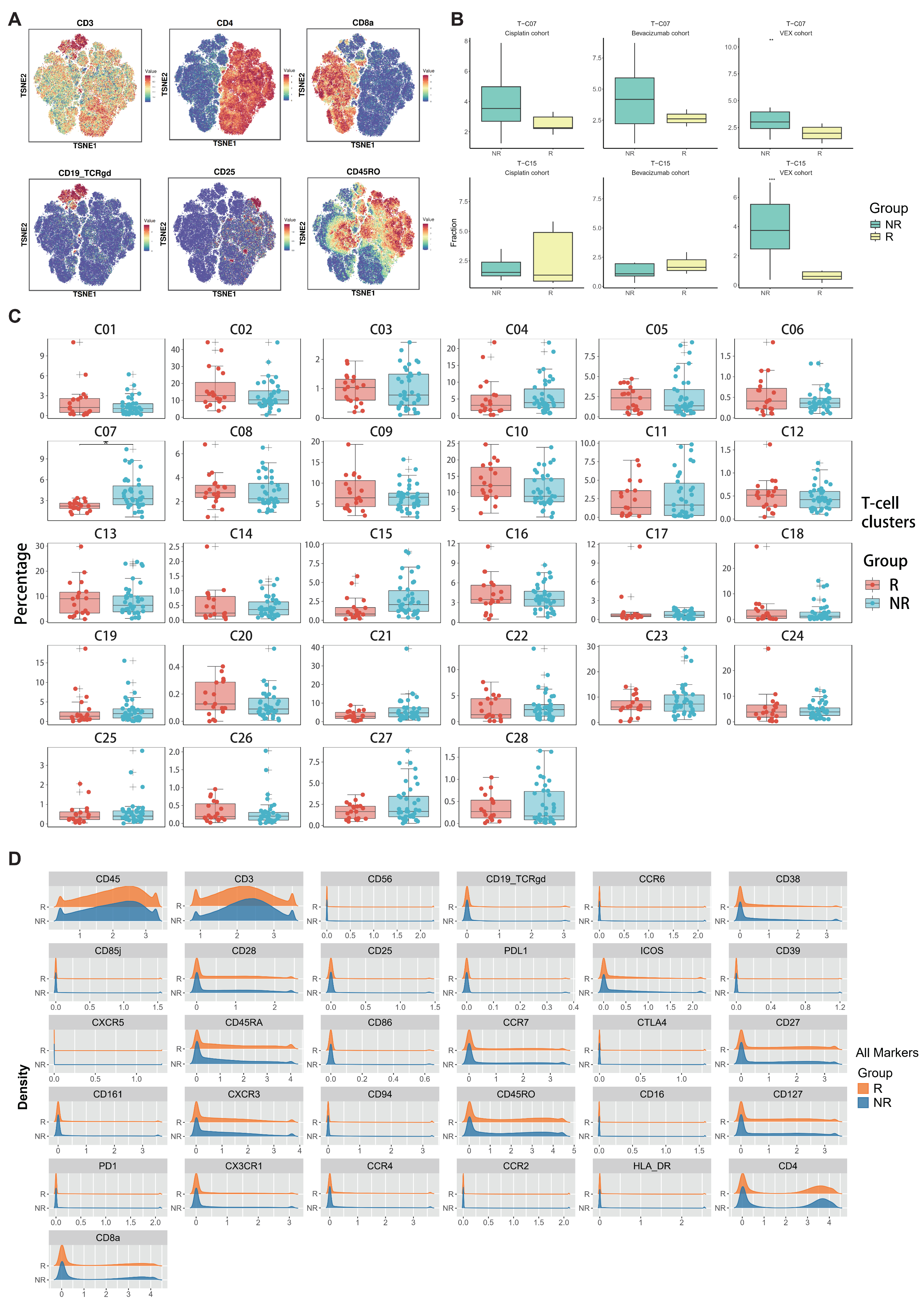


**Figure S3. Dynamic analysis of T-cell compartment, related to Figure 3.**


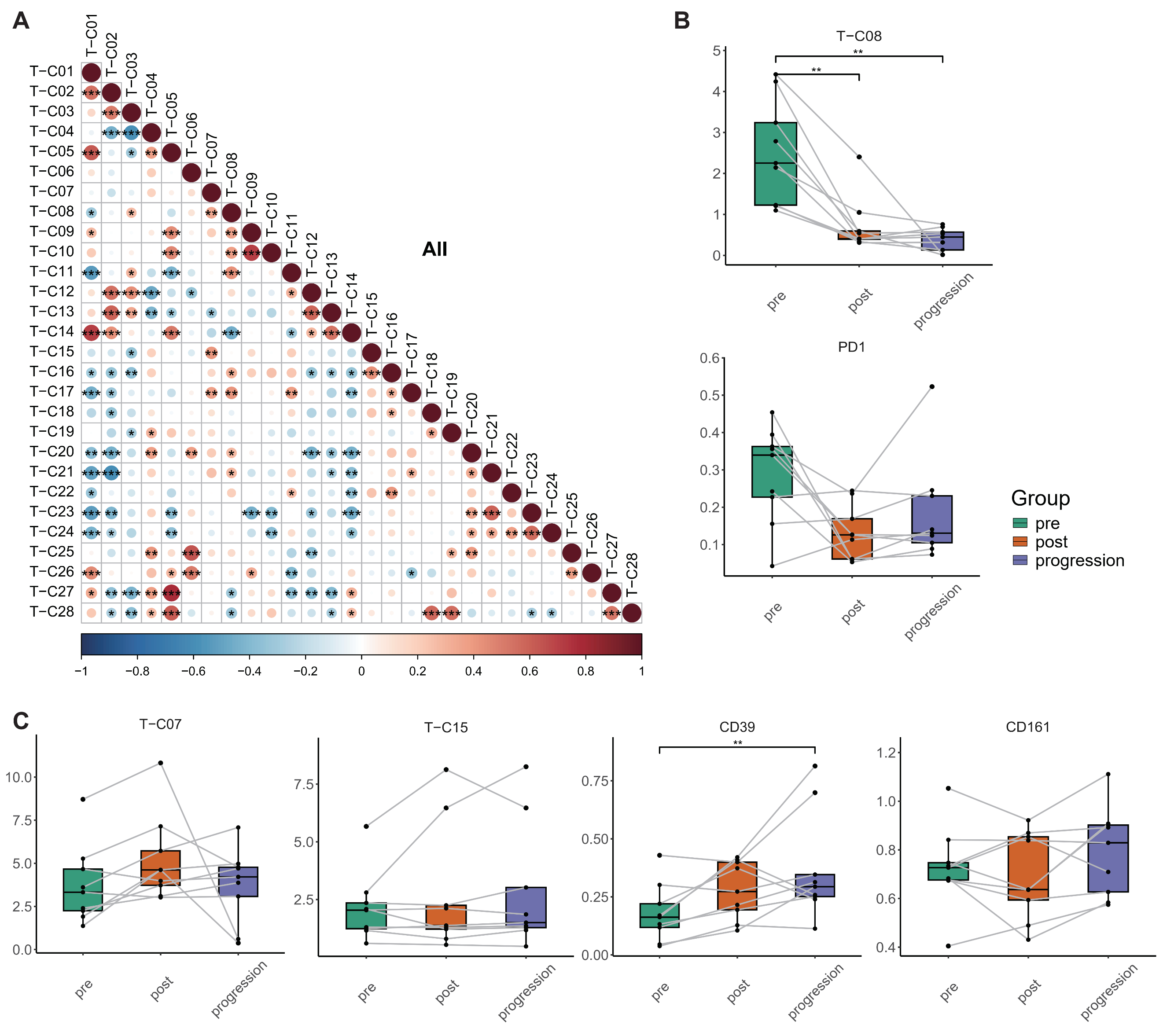


**Figure S4. In-depth analysis of myeloid cell compartment, related to Figure 4.**


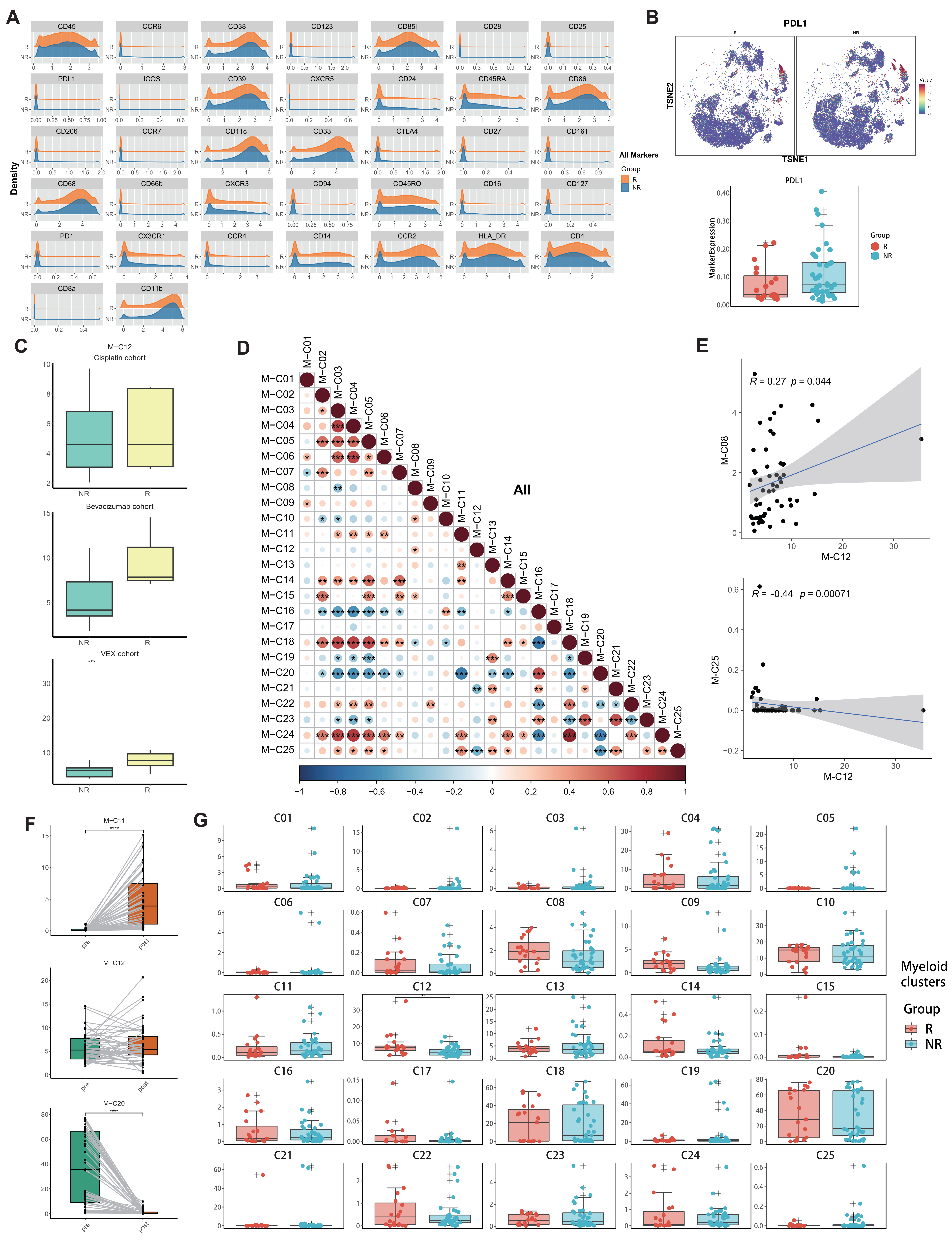


**Figure S5. Model test, related to Figure 5.**


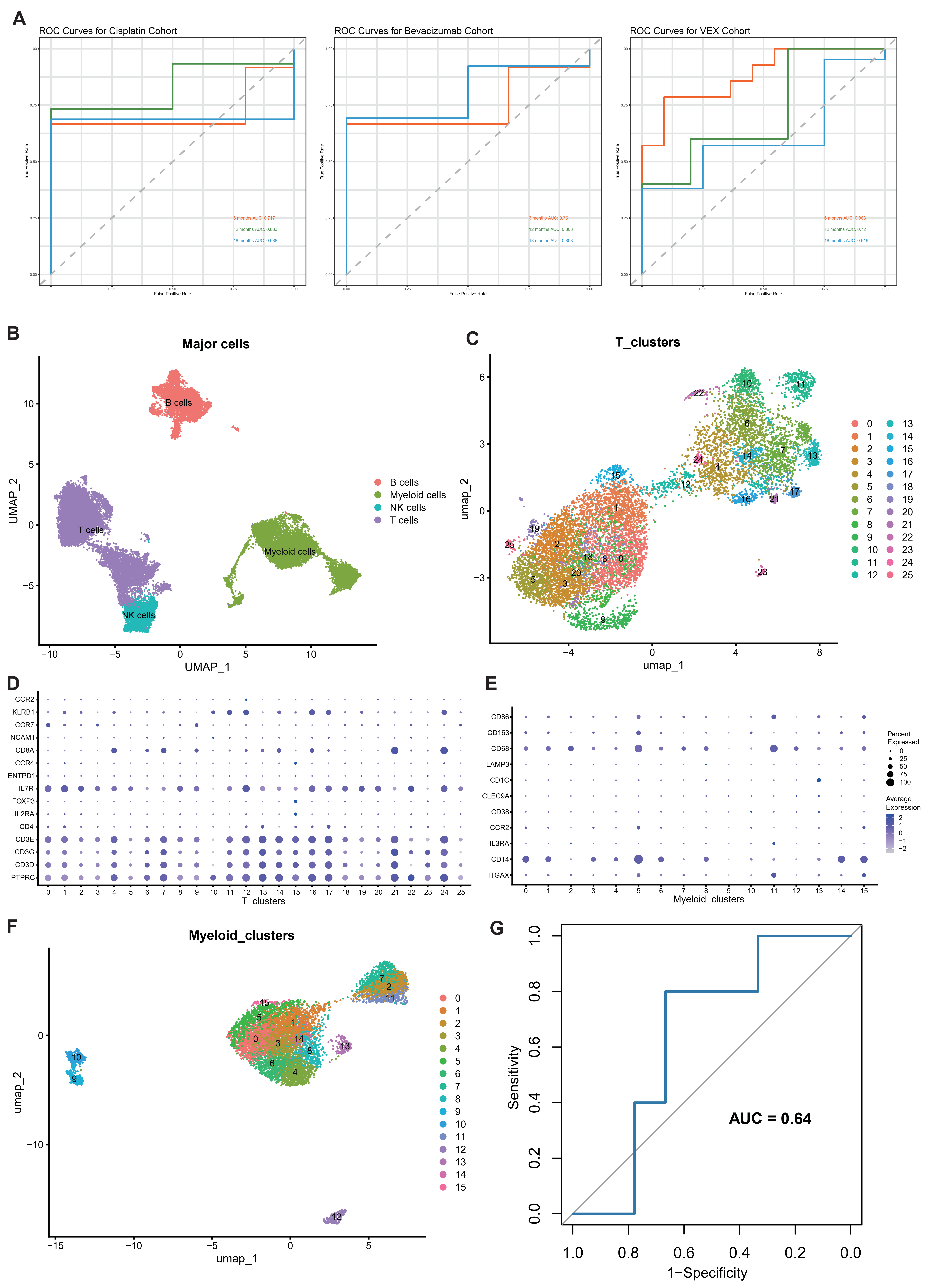
 *External validation utilized an independent single-cell RNA sequencing dataset (GSE189125) from the Gene Expression Omnibus (GEO) database (https://www.ncbi.nlm.nih.gov/geo/).*
