# Supplementary material for: Predictive circulating biomarkers of the response to anti‐PD‐1 immunotherapy in advanced HER2 negative breast cancer
Source: Clin Transl Med. 2025 Feb 25;15(3):e70255. doi: 10.1002/ctm2.70255 (PMC11859116; doi:10.1002/ctm2.70255)
Supplement: Supplementary file 3 — Supporting Information [file CTM2-15-e70255-s002.docx]

**Predictive Circulating Biomarkers of the Response to Anti-PD-1 Immunotherapy in Advanced HER2 Negative Breast Cancer**

Yuhan Wei, Hewei Ge, Yalong Qi, Cheng Zeng, Xiaoying Sun, Hongnan Mo, Fei Ma

**Supplementary method for external validation**

To validate our model, we utilized an independent single-cell RNA sequencing dataset (GSE189125) from the Gene Expression Omnibus (GEO) database (https://www.ncbi.nlm.nih.gov/geo/), which focused on a cohort of patients undergoing immunotherapy.

Specifically, after downloading the data, we removed low-quality cells with less than 300 genes detected, more than 10,000 genes detected, or fewer than 400 unique molecular identifiers (UMIs). The default parameters of Seurat were used unless otherwise indicated. For the clustering of all cell types, 2,000 variable genes were identified, and principal component analysis was applied to the dataset to reduce dimensionality. The 50 most informative principal components were used for clustering and UMAP visualization. Afterward, the parameter resolution to 1.2 was set for the “Find Clusters” function for clustering analyses. Following initial unsupervised clustering, we annotated cell clusters using typical immune cell markers, identifying major types including T cells, NK cells, B cells, and myeloid cells. We then isolated T cells and myeloid cell subpopulations for a second round of unsupervised clustering (resolution 1.6) to obtain a high-resolution map of patient immune cell populations. To validate our model, we examined gene expression of markers in subpopulations corresponding to our CyTOF findings, identifying cell subsets of interest. Finally, the frequencies of these identified cell subsets were input into our model for validation, using the area under the curve (AUC) values of treatment response as the evaluation metric.

Clinical features for each sample were obtained from the supplementary files supplied by the original article^[1]^. Figures visualizing of the single-cell RNA sequencing findings were generated using a combination of Seurat^[2]^ and ggplot2 R packages.

**References**

1. Lozano AX, Chaudhuri AA, Nene A, Bacchiocchi A, Earland N, Vesely MD, et al. **T cell characteristics associated with toxicity to immune checkpoint blockade in patients with melanoma**. *Nat Med* 2022; 28(2):353-362.

2. Hao Y, Hao S, Andersen-Nissen E, Mauck WM, Zheng S, Butler A, et al. **Integrated analysis of multimodal single-cell data**. *Cell* 2021; 184(13):3573-3587.e3529.
